# High‐Viability Circulating Tumor Cells Sorting From Whole Blood at Single Cell Level Using Laser‐Induced Forward Transfer‐Assisted Microfiltration

**DOI:** 10.1002/advs.202414195

**Published:** 2025-01-27

**Authors:** Qingmei Xu, Yuntong Wang, Songtao Dou, Yang Xu, Zhenhe Xu, Han Xu, Yi Zhang, Yanming Xia, Ying Xue, Hang Li, Xiao Ma, Kunlong Zhang, Huan Wang, Fengzhou Ma, Qi Wang, Bei Li, Wei Wang

**Affiliations:** ^1^ School of Integrated Circuits Peking University Beijing 100871 China; ^2^ Department of Electrical Engineering Taiyuan Institute of Technology Taiyuan 030008 China; ^3^ Changchun Institute of Optics Fine Mechanics and Physics Chinese Academy of Sciences Changchun 130033 China; ^4^ University of Chinese Academy of Sciences Beijing 100049 China; ^5^ Cancer Translational Medicine Research Center The Second Affiliated Hospital of Dalian Medical University Dalian 116027 China; ^6^ Department of Respiratory Medicine The Second Affiliated Hospital of Dalian Medical University Dalian 116027 China; ^7^ State Key Laboratory of Bioreactor Engineering Shanghai Frontiers Science Center of Optogenetic Techniques for Cell Metabolism East China University of Science and Technology Shanghai 200237 China; ^8^ Guangzhou National Laboratory Guangzhou 510320 China; ^9^ Hooke Laboratory Changchun 130033 China; ^10^ Hangzhou Branemagic Medical Technology Co. Ltd. Hangzhou 310021 China; ^11^ National Key Laboratory of Advanced Micro and Nano Manufacture Technology Beijing 100871 China; ^12^ Beijing Advanced Innovation Center for Integrated Circuits Beijing 100871 China

**Keywords:** circulating tumor cells (CTCs), laser‐induced forward transfer, microfiltration, single‐cell culture, single‐cell RNA sequencing

## Abstract

The efficient isolation and molecular analysis of circulating tumor cells (CTCs) from whole blood at single‐cell level are crucial for understanding tumor metastasis and developing personalized treatments. The viability of isolated cells is the key prerequisite for the downstream molecular analysis, especially for RNA sequencing. This study develops a laser‐induced forward transfer ‐assisted microfiltration system (LIFT‐AMFS) for high‐viability CTC enrichment and retrieval from whole blood. The LIFT‐compatible double‐stepped microfilter (DSMF), central to this system, comprises two micropore layers: the lower layer's smaller micropores facilitate size‐based cell separation, and the upper layer's larger micropores enable liquid encapsulating captured cells. By optimizing the design of the DSMFs, the system has a capture efficiency of 88% at the processing throughput of up to 15.0 mL min^−1^ during the microfilter‐based size screening stage, with a single‐cell yield of over 95% during the retrieval stage. The retrieved single cells, with high viability, are qualified for ex vivo culture and direct RNA sequencing. The cDNA yield from isolated CTCs surpassed 4.5 ng, sufficient for library construction. All single‐cell sequencing data exhibited Q30 scores above 95.92%. The LIFT‐AMFS shows promise in cellular and biomedical research.

## Graphical abstract

A laser‐induced forward transfer‐assisted microfiltration system is developed for single rare cell sorting from undiluted whole blood. The isolated cell has a high viability, enabling successful ex vivo cell culture and efficient single‐cell RNA sequencing. The technology offers a robust and versatile tool for advancing single‐CTC research, providing significant opportunities for both clinical applications and a deeper understanding of metastasis mechanisms.

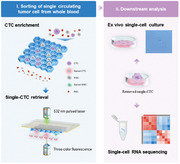

## 1 Introduction

Circulating tumor cell (CTC) is a rare and heterogeneous population of cancer cells that circulate in the bloodstream,^[^ ^1^, ^2^, ^3^ ^]^ which are significant biomarkers in cancer metastasis detection^[^ ^4^, ^5^ ^]^ and personalized medicine development.^[^ ^6^ ^]^ Traditional methods for CTC analysis, e.g., enumeration or staining,^[^ ^7^, ^8^ ^]^ have contributed valuable insights on evaluating metastatic potential and clinical implications.^[^ ^3^ ^]^ However, few technologies can characterize CTCs at the molecular level beyond mere enumeration.^[^ ^9^ ^]^ To gain deeper insights into the functions of CTCs, single‐CTC profiling techniques have emerged as vital tools for investigating tumor heterogeneity.^[^ ^10^ ^]^ Efficient sorting of single CTC with high viability from complex blood environments is a prerequisite for effective single‐cell analysis.^[^ ^11^, ^12^, ^13^ ^]^ The single CTC with high viability could not only facilitate personalized oncology by ex vivo culture but also provide exciting opportunities to understand the metastasis process through single‐cell RNA sequencing (RNA‐Seq).^[^ ^14^, ^15^ ^]^

For years, significant progress has been made in developing single‐cell sorting techniques, including fluorescence‐activated cell sorting,^[^ ^16^, ^17^ ^]^ magnetic‐activated cell sorting,^[^ ^18^ ^]^ and high‐throughput approaches based on microwell^[^ ^19^ ^]^ or microdroplet^[^ ^20^ ^]^ technologies. These techniques have emerged to facilitate the molecular characterization of CTCs burgeon at the single‐cell level. However, these methodologies are inadequate for handling samples with rare cells or minimal amounts of cell input samples. Accordingly, integrated platforms have been developed to retrieve single‐CTC from whole blood.^[^ ^21^, ^22^ ^]^ Specifically, a CTC pre‐enrichment technology is first adopted to remove many background cells, and then the rare tumor cells are picked up from the captured cells. The presorting technologies for CTC enrichment have been mainly fulfilled based on the CTC physical properties, e.g., size,^[^ ^23^ ^]^ deformability,^[^ ^24^ ^]^ electrical property,^[^ ^25^ ^]^ or based on biological affinities, e.g., immune magnetic^[^ ^26^ ^]^ and affinity chromatography.^[^ ^27^ ^]^ The retrieval technologies of the single‐CTC include mechanical micromanipulation,^[^ ^28^ ^]^ laser capture microdissection (LCM),^[^ ^29^ ^]^ and laser‐induced forward transfer (LIFT).^[^ ^30^ ^]^ In several studies, CTCs were enriched by sized‐based microfilter^[^ ^31^ ^]^ or microfluidic pre‐label devices,^[^ ^32^ ^]^ and single‐CTC was then picked up by the micromanipulator manually. The conventional method of isolating single‐CTC through micromanipulation provides a high degree of accuracy and minimizes the loss of samples; however, it requires substantial time and effort, which restricts its practical use in clinical.^[^ ^33^ ^]^ Furthermore, the LCM technique, which is a direct contact method, has recently coupled with sized‐based microfiltration^[^ ^34^ ^]^ or microfluidic CTC positive enrichment chips^[^ ^35^ ^]^ for whole exome research. The potential problems could be cell damage and low throughput induced by ultraviolet laser.^[^ ^36^ ^]^

In contrast, LIFT technology is a promising non‐contact method to process CTCs after pre‐enrichment, in which a pulsed laser generates microbursts that convert opto energy into mechanical energy, causing the target sample to be ejected onto a receiving substrate;^[^ ^37^ ^]^ but it is limited by low capture throughput and detection accuracy in liquid. The integration of biocompatible and optically transparent polymers, such as SU‐8 and Parylene C, with LIFT can enhance its effectiveness. As one of the methods to retrieve single‐CTC, EpCAM‐coated SU8 micropillars were employed to capture the CTCs and a near‐infrared light beam to isolate the microcolumns with target cells, which maintains the integrity of cells.^[^ ^38^ ^]^ However, this method carries risks of multiple cells being captured on a single micropillar and nonspecific binding. In addition, although the potential of Parylene C‐based single‐layer microfilters for capturing CTCs from undiluted whole blood samples has been demonstrated,^[^ ^39^ ^]^ the high surface tension of these microfilters hindered the efficient retrieval of the captured cells under the pulsed laser, which caused them to slide along the surface rather than being ejected (Figure S1, Supporting Information).^[^ ^40^ ^]^ Therefore, a multifunctional LIFT‐based system, capable of sorting single‐CTCs from whole blood with high viability and high throughput simultaneously, is urgently needed for CTCs profiling at the single‐cell level.

Here, this work develops a LIFT‐assisted microfiltration system (LIFT‐AMFS), which seamlessly integrates the double‐stepped microfilter (DSMF) with LIFT technology. This innovative system is designed to efficiently sort single rare cells with high viability from undiluted whole blood at high throughput. The LIFT‐compatible DSMFs, as the core component of the system, enable effective enrichment and retrieval of single cell, making them well‐suited for downstream analyses that require high cell viability. As a proof of concept, single‐cell ex vivo culture and high‐quality single‐cell RNA‐Seq are simultaneously conducted to validate the viability of the retrieved cells. The proposed LIFT‐AMFS is an innovative method for high‐viability single rare cell retrieval that enhances both isolation efficiency and accuracy, providing a valuable tool for tumor biology research at the single‐cell level.

## 2 Results and Discussion

### 2.1 Working Principle of the LIFT‐AMFS

The LIFT‐AMFS for single‐CTC retrieval comprises two components: the single‐cell sorting chip and the LIFT platform. The single‐cell sorting chip includes a DSMF and a glass substrate coated with a sacrificial layer of TiN. The 50‐nm‐thick TiN layer can efficiently absorb energy, minimizing potential cell damage. Following cell enrichment, the DSMF is bonded to the glass substrate through plasma treatment of the surface, which ensures proper alignment and consistent spacing between the glass substrate and the microfilter, facilitating accurate cell picking. The LIFT platform integrates an ejection sorting system, an imaging recognition system, two 3D motorized stages, and a receiving system (Figure S2, Supporting Information). The two motorized stages are computer‐controlled: one is used to locate target cells and the other to position a 96‐cell culture plate to receive the retrieved cells.

**Figure 1:**
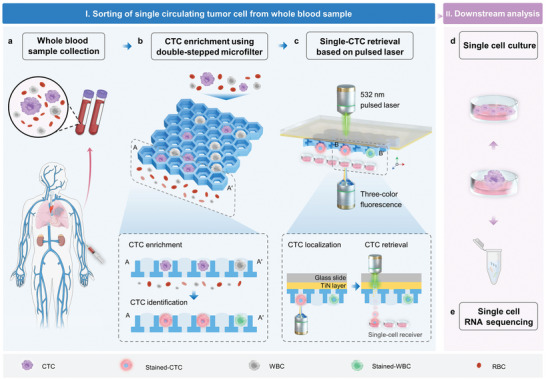
Schematic overview of single‐CTC enrichment and retrieval via the LIFT‐AMFS. a) A simulated sample is prepared by spiking rare tumor cells into whole blood. b) CTCs are enriched using the DSMF under gravity. In situ live cell staining is performed to identify CTCs (CK7+/CD45‐/Hoechst+) from nonspecifically captured WBCs (CK7‐/CD45+/Hoechst+). c) The stained‐CTCs are precisely located and released using a nano‐second pulsed laser that vaporizes the TiN layer. d) The retrieved cell is cultured and proliferated ex vivo within the cell receiver. e) RNA from the target cell is extracted and processed following the SMART‐Seq II sequencing protocol for comprehensive single‐cell transcriptome analysis.

The LIFT‐AMFS workflow (**Figure** **1**) comprised three main steps: 1) CTC pre‐enrichment, 2) single‐cell retrieval, and 3) downstream viability assessment via both single‐cell ex vivo culture and RNA‐Seq. Lung cancer cell lines with high expression of CK7 protein were used to mimic CTCs in clinical blood samples (Figure 1a). Specifically, the CTCs were first captured and trapped within individual micropores under gravity (Figure 1b). Subsequently, a live cell staining protocol was established to distinguish CTCs (CK7+, CD45‐, Hoechst+) from nonspecifically captured white blood cells (WBCs) (CK7‐, CD45+, Hoechst+) (Figure 1b; Figure S3, Supporting Information). Following molecular labeling, the DSMF was disassembled and transferred onto a titanium nitride (TiN)‐coated glass substrate. By employing two automated stages facing each other with a synchronized pulse laser, the target cells were identified and retrieved at high throughput and transferred to the cell receiver for immediate downstream analysis (Figure 1c). Finally, single‐cell ex vivo culture (Figure 1d) and single‐cell RNA‐Seq (Figure 1e) were simultaneously conducted on the retrieved cells. Typically, the entire process was completed within 90 min (Figure S4, Supporting Information).

The designed system and LIMO‐Seq^[^ ^38^ ^]^ both employ a nanosecond‐pulsed laser to single out individual CTC, allowing for meticulous single‐cell handling with a reduced risk of damage, thus maintaining cell viability throughout the procedure. What sets this system apart is the strategic use of large and small micropores in the DSMF device, which boosts the specificity of single‐cell capture and substantially reduces the chances of nonspecifically trapping multiple cells. Moreover, this system offers a label‐free CTC pre‐enrichment approach that leverages size differences, enhancing the likelihood of capturing a diverse range of CTC phenotypes.

### 2.2 Design of DSMF for the CTC Enrichment

The innovative design of the Parylene C‐based DSMF, compatible with LIFT, is crucial for the efficient enrichment and retrieval of single CTC from whole blood. A dense‐packed hexagonal micropore array maximizes porosity while ensuring structural stability. The developed microfilter features two layers of micropores with different sizes and thicknesses, each serving a specific function in the CTC isolation process. The upper layer contains larger micropores (diagonal lengths (D) of 15, 20, and 25 µm) to match the typical CTC diameter range, ensuring single‐cell capture and encapsulating the liquid layer surrounding the trapped cells. This liquid layer reduces surface tension, significantly minimizing energy requirements during LIFT, thereby preserving cell viability. The thickness of the upper layer is 20 µm to ensure complete entrapment of cells while maintaining structural integrity. The lower layer, with smaller micropores (diagonal lengths (d) of 8 and 10 µm), focuses on size‐based separation and enrichment. Its 10 µm thickness further supports cell viability by minimizing transmembrane pressure. To prevent non‐specific adhesion, the edge‐to‐edge spacing (S) of the micropores in the upper layer is 4 µm, smaller than typical blood cells.

**Figure 2:**
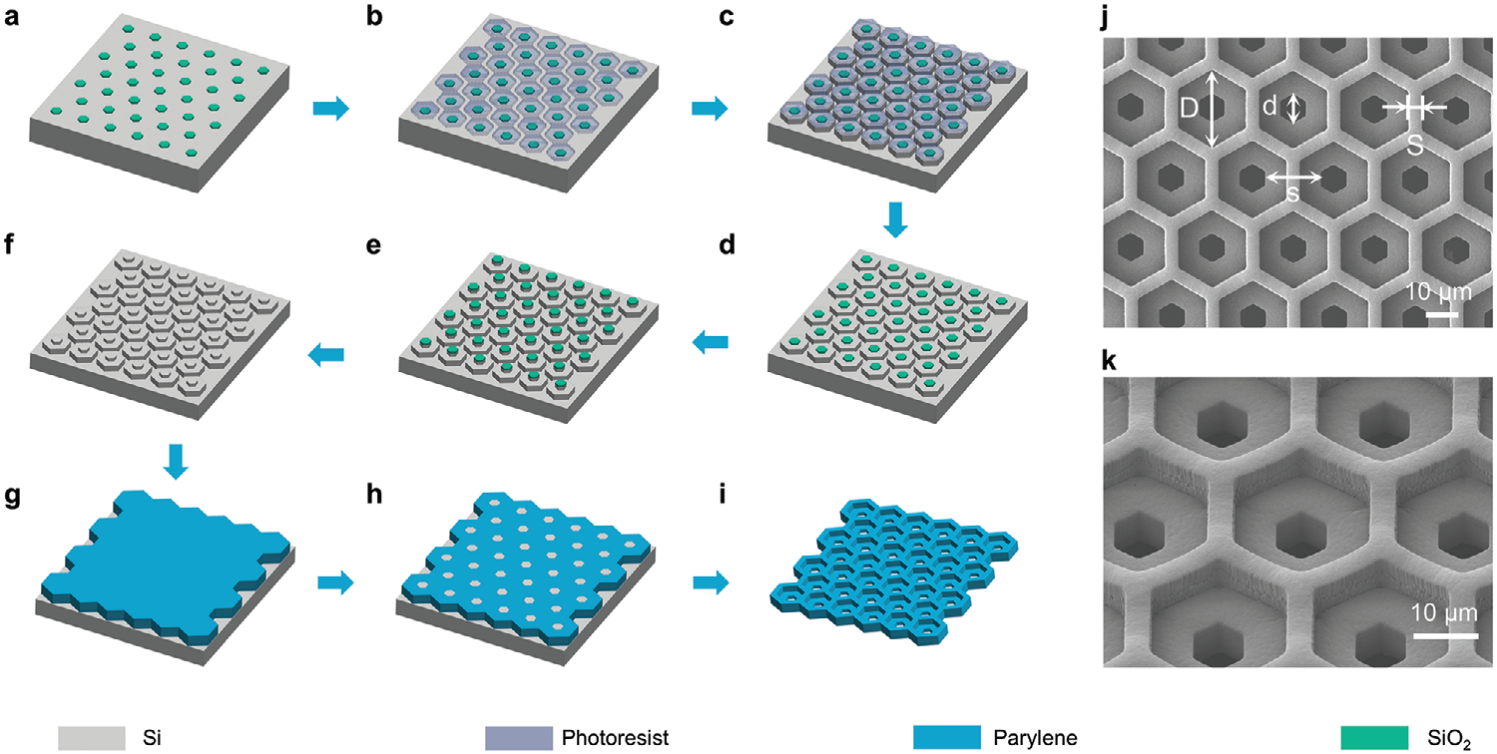
The workflow developed for the fabrication of the DSMF. a) The small micropores of the lower layer are defined by RIE. b) The large micropores of the upper layer are defined by photolithography patterning. c) The large micropores of the upper layer are obtained via 1st DRIE. d) The photoresist is removed in preparation for the fabrication of the small micropores. e The small micropores of the lower layer are obtained via the 2nd DRIE. f) The silicon oxide is removed. g A Parylene C layer with a specified thickness is deposited via chemical vapor deposition. h) RIE is conducted to remove the Parylene C layer that extends beyond the height of the micropillars. i) The DSMF is released via wet etching. j,k) SEM images of the fabricated DSMF.

The DSMF was meticulously engineered for cell enrichment and retrieval, employing a two‐layer silicon molding technique based on Parylene C. The fabrication procedure for the DSMF is summarized in **Figure** **2**. First, the small apertures of the lower layer were defined using reactive ion etching (RIE) on silicon oxide (Figure 2a). Second, the large apertures of the upper layer, which were situated within the small apertures, were defined via photolithography patterning (Figure 2b). Third, the template of the upper layer was fabricated through deep reactive ion etching (DRIE) on a Si wafer to form a hexagonal micropillar array with a height of 20 µm (Figure 2c). Subsequently, the photoresist was removed (Figure 2d) and the lower‐layer template containing a hexagonal micropillar array with a height of 10 µm was obtained via DRIE (Figure 2e). Next, the silicon oxide was removed (Figure 2f). A Parylene C layer with a specified thickness was deposited onto the Si template using a Parylene deposition instrument (SCS, PDS2010) (Figure 2g). Afterward, the RIE of the deposited Parylene C layer was performed to expose the top of the silicon pillars (Figure 2h). Finally, the prepared Parylene C‐based microfilter was released from the Si substrate after immersion in an HNA (HF: HNO_3_: HAc = 5: 7: 11, v/v) solution (Figure 2i).

The typical scanning electron microscopy (SEM) images of the microfilter (design 6 in Table S1, Supporting Information) are presented in Figure 2j,k. The overall size of the microfilter was 17 mm × 17 mm, with an effective enrichment area larger than 13 mm × 13 mm, providing a substantial area for CTC capture and processing. Compared to single‐layer microfilters,^[^ ^39^ ^]^ the proposed DSMF effectively overcomes the problems related to high surface tension in single‐layer microfilters, which is a LIFT‐compatible device.

### 2.3 Enrichment of CTC Through the DSMF

**Figure 3:**
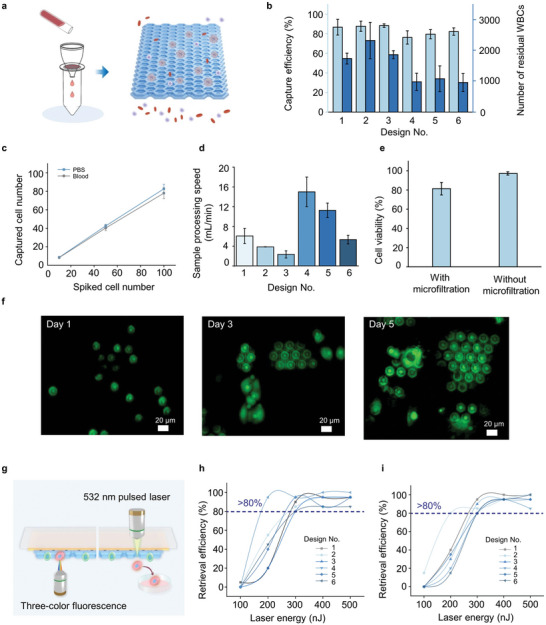
Enrichment and retrieval performance of LIFT‐AMFS. a) Schematic of CTC enrichment via the DSMF. b) The capture efficiency of PC9‐GFP cells (light blue bars) and the residual counts of WBCs (dark blue bars) in whole blood samples using various microfilters are depicted. c) The capture efficiencies at different spiked cell concentrations ranging from 10 to 100 cells per mL. d) Sample processing speed of the DSMF. e) Viability of PC9‐GFP cells with and without enrichment using the DSMF. f) Fluorescence micrographs of PC9‐GFP cells (visualized by Calcein‐AM staining) on the microfilter over several days (days 1, 3, and 5), confirming its ability to support in situ cell culture. g) Schematic illustration of the CTC retrieval process using the LIFT‐AMFS. h) Retrieval efficiency in PBS at different energy levels. i) Retrieval efficiency in whole blood at different energy levels. Data are presented as mean ± SD for (b–e), *n* = 3 for (b–e), *n* = 20 for (h and i).

The capture efficiency of the DSMF was evaluated using GFP‐expressing PC‐9 (PC9‐GFP) cells spiked into phosphate‐buffered saline (PBS) and whole blood from healthy volunteers (**Figure** **3**a). Six microfilters with different micropore sizes exhibited high recovery (80.1 ± 13.5% to 87.4 ± 2.11%) when processing 10 mL PBS, demonstrating their capability to effectively handle large‐volume samples (Figure S5, Supporting Information). The microfilters with a micropore size of 8 µm in the lower layer exhibited superior recovery compared to those with a micropore size of 10 µm, suggesting that smaller micropores were more effective at capturing target cells.

The capability of the DSMF to isolate tumor cells from whole blood was further assessed using a spiked sample containing ≈500 PC9‐GFP cells/mL. The capture efficiency of the six microfilters ranged from 76.2 ± 6.7% to 88.3 ± 1.8% (Figure 3b). After filtration, the residual WBCs on the DSMF averaged 1976 for the micropore size of 8 µm and 1009 for the 10 µm one. While the 8 µm micropore‐sized microfilter captured a greater number of WBCs, the subsequent single‐cell picking procedure, which operates at the single‐cell resolution level, remained unaffected by the presence of these additional WBCs. Ultimately, the DSMF with the smaller micropore size (8 µm) was selected to ensure efficient capture of CTCs with varied diameters. To evaluate the capture efficiency for small numbers of CTCs, different concentrations (ranging from 10 to 100 cells per mL) of PC‐9 were spiked into both PBS and blood under the optimal capture conditions. Consistent recovery was observed at various numbers of spiked cells as low as 10 cells per mL (Figure 3c). As a clinical validation of this device, we tested the invented DSMF with blood samples collected from five lung cancer patients. Immunostaining of CK7 and CD45 was performed to confirm the presence of CTCs and WBCs, respectively. The detection of CTCs from all of the five whole blood samples was positive (Figure S6, Supporting Information), although there was variance in the counts of verified CTCs for different lung cancer patients (Table S2, Supporting Information).

The processing speed of undiluted whole blood through the DSMF was examined across designs with different porosities (designs 1–6 in Table S1, Supporting Information). The microfilter with a micropore size of 10 µm exhibited higher processing speeds (5.3 ± 0.9 to 15.0 ± 3.0 mL min^−1^) compared to that with a micropore size of 8 µm (2.3 ± 0.7 to 6.1 ± 1.5 mL min^−1^) (Figure 3d). The processing speed was mainly affected by the porosity of the microfilter, particularly that of the lower layer. A demonstration of processing 6 mL of whole blood (using design 4 from Table S1, Supporting Information) is presented in Movie S1 (Supporting Information). The rapid processing speed, achieved without requiring external driving equipment, ensures the viability of the captured cells and lays a solid foundation for subsequent cell culturing and RNA‐Seq.

The viability of cells captured using the DSMF (design 5 in Table S1, Supporting Information) was assessed using a Calcein AM/ethidium homodimer‐1 fluorescent assay. The captured cells reached a high viability rate of 81.3 ± 6.5%, highlighting the good biocompatibility of the microfilter and its ability to minimize apoptosis while preserving high‐quality genomic information (Figure 3e). Furthermore, an in situ culture of the captured cells on the microfilter was conducted, exhibiting robust cell adhesion and proliferation on days 1, 3, and 5 (Figure 3f). These findings validate the biocompatibility of the DSMF and its effectiveness in preserving the viability and integrity of the captured cells.

### 2.4 Retrieval of Single Rare Cell through LIFT‐ AMFS

To determine the optimal laser energy for sorting a single CTC, different laser energy levels (100‒500 nJ) were applied to the cells captured by the DSMF in both PBS and whole blood samples (Figure 3g). The results in Figure 3h,i exhibited a significant improvement in the retrieval efficiency, achieving ≥80% when the laser energy was increased from 200 to 300 nJ, regardless of whether the sample was PBS or whole blood. Moreover, the retrieval efficiency remained consistently high (80–100%) at laser energy levels above 300 nJ. The single‐cell yield of the proposed system for retrieving target cells from whole blood was ≈100% (Table S3, Supporting Information). This can be attributed to the innovative design of the DSMF and its synergistic integration with the LIFT platform.

**Figure 4:**
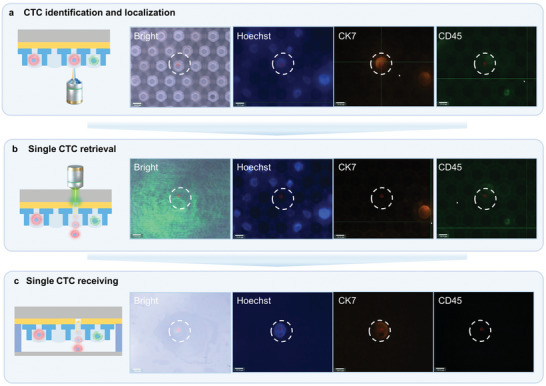
Micrograph images of the single‐cell retrieval process using the LIFT‐AMFS. a) Identification and localization of the CTC (CK7+/CD45‐/Hoechst+) in a micropore via bright‐field and fluorescence microscopy. b) Retrieval of a single CTC via a pulsed laser, which targets the TiN layer to propel the cell out of the micropore. c) Successful reception of the single cell in the cell receiver (a 0.17 mm‐thick glass coverslip), with the cell maintaining its morphology intact. The scale bar in each image represents 10 µm.

**Figure** **4** exhibits representative micrographs of the CTC retrieval from undiluted whole blood under a laser energy of 300 nJ; the dynamic process is demonstrated in Movie S2 (Supporting Information). First, the captured cell was identified via both bright field and fluorescence microscopy (Figure 4a). Subsequently, a laser pulse was applied to the target cell (CK7+/CD45‐/Hoechst+) trapped within the micropore (Figure 4b). The TiN layer absorbed the energy, effectively pushing the cell into the receiver (Figure 4c). The retrieved target cell maintained its morphology intact in the cell receiver (a 0.17 mm‐thick glass coverslip), without any noticeable adverse effects on the cells. Considering both the retrieval efficiency and the morphology of the retrieved cells, the optimal retrieval energy was ascertained to be 300 nJ. The sorting process is exceptionally rapid, with the laser pulse for only 5 ns and a total sorting time of 2 s for each cell. Moreover, the retrieved cell is immediately transferred to a lysis solution for RNA extraction. This minimal processing time ensures the RNA transcriptome remains stable, preserving RNA integrity for downstream analyses.

### 2.5 Downstream Analysis of the Retrieved Single‐Cell

The viability of a single CTC is critical for conducting single‐cell RNA‐Seq to uncover the inherent heterogeneity of tumor cells. For a successful single‐cell RNA‐Seq, the purity and viability of the isolated CTCs are paramount. To comprehend the impact of the retrieval process on cell viability, numerical simulations were performed to analyze the temperature distribution induced by the interaction of the pulsed laser energy with the TiN layer. A double‐stepped micropore model was employed to simulate temperature variation at varying distances from the metal surface. As shown in Figure S7 (Supporting Information), the position 5.1 µm from the metal surface consistently remained below 37 °C, which was optimal for cell survival. Given the total micropore thickness of 30 µm and the typical cell diameter of ≈15 µm, cells subjected to gravity during chip inversion were positioned well beyond this critical distance, indicating minimal thermal injury on cell integrity and endorsing the viability of the subsequent single‐cell culture process. In contrast, the conventional LIFT system, i.e., without DMSF, revealed that the pulsed laser caused a rapid increase in the surface temperature of the TiN, far exceeding the survival threshold of cells (Figure S8, Supporting Information). Consequently, in the LIFT‐AMFS system, despite the intense instantaneous energy delivered by LIFT to the metal surface, the temperature in the vicinity of the cells remains low, ensuring high cell viability during LIFT operation. This is attributed to the intelligent structural design of the DSMF, which keeps cells at a safe distance from the site of action.

**Figure 5:**
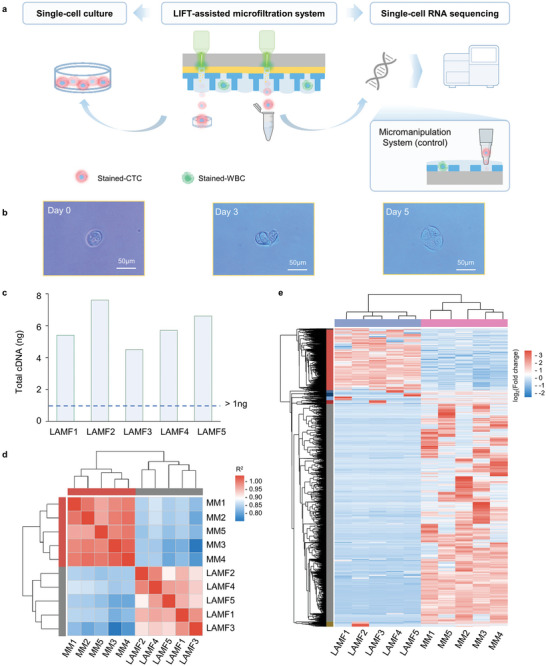
Downstream analysis of the single retrieved cell from the LIFT‐AMFS. a) Overall workflow for conducting single‐cell culture and single‐cell RNA‐Seq after retrieving single CTC via the LIFT‐AMFS. b) Optical microscopy images of retrieved tumor cells on days 0 (immediately after microfiltration), 3, and 5. c) Total cDNA of single‐cell RNA reverse transcription‐amplified. The dashed line denotes the minimum cDNA input required for constructing a cDNA library and performing sequencing. d) Heat map of sample correlation between single CTC processed using the LIFT‐AMFS (LAMFX, X = 1, 2, 3, 4, 5) and the micromanipulation system (MMX, X = 1, 2, 3, 4, 5). e) Heat map of differential gene expression in the LIFT‐AMFS (LAMFX, X = 1,2,3,4,5) and the micromanipulation system (MMX, X = 1,2,3,4,5).

To validate these simulation predictions, the viability of the retrieved cells was assessed via ex vivo culture experiments (**Figure** **5**a). Regardless of the tumor cell concentration in the sample, the retrieved cells exhibited strong adhesion and proliferation in the cell receiver, confirming their high post‐retrieval viability (Figure 5b; Figure S9, Supporting Information). Overall, this viability underscores the suitability of the LIFT‐AMFS for single‐cell RNA‐Seq.

To demonstrate the feasibility of using LIFT‐AMFS to isolate viable single CTC for downstream molecular assays, single‐cell RNA‐Seq was conducted on the retrieved cells sorted using the LIFT‐AMFS (LAMFX, X = 1, 2, 3, 4, 5). According to the Nextera XT DNA Library Prep Kit datasheet, a minimum of 1 ng cDNA input is required for cDNA library construction and sequencing. As presented in Figure 5c, the total cDNA from the retrieved cells exceeded 4.5 ng, surpassing the requirement and enabling successful RNA‐Seq. The reverse transcription amplification of single‐cell RNA confirmed the effectiveness of the proposed system.

Subsequently, the amplified RNA from the target cells was used to construct cDNA libraries, which were sequenced to yield over 44 million clean reads per cell, with a base quality Q30 score above 95.92% and a Guanine‐Cytosine (GC) content of ≈50% (Table S4, Supporting Information). These metrics indicate the high quality of the single‐cell data, ensuring reliable downstream analysis. The correlation analysis among biological replicates demonstrated strong reproducibility and consistent transcript expression patterns (Figure 5d). In addition, differential gene expression analysis revealed a significant overlap in the highly expressed genes in the LAMF group and the MM group, respectively (Figure 5e). The gene expression patterns within each group exhibited strong intra‐group correlation, demonstrating the consistency and biological relevance of the retrieved cells.

Next, LIFT‐AMFS was compared with traditional micromanipulation techniques for single‐cell analysis and characterization.^[^ ^28^ ^]^ After sorting cells using the DSMF (Figure 5a), single‐cell RNA‐Seq was performed using both the LIFT‐assisted system and a micromanipulation system (MMX, X = 1, 2, 3, 4, 5). While both methods successfully executed single‐cell RNA‐Seq, the sample correlation and differential gene expression analyses revealed distinct populations between the cells retrieved by the two systems. This discrepancy is primarily attributed to differences in the retrieval process. The micromanipulation system involves direct physical contact with cells via a micropipette, which can induce mechanical stress, alter gene expression, and potentially cause cell damage.^[^ ^33^, ^41^, ^42^ ^]^ In contrast, the proposed LIFT‐AMFS system employs a noncontact retrieval method that minimizes physical stress and cell perturbation. Additionally, the rapid retrieval time of 1–2 s for each target cell in the LIFT‐AMFS system further reduces the risk of transcriptomic changes compared to the prolonged operation time (≈10 min for each target cell) of micromanipulation. As a result, the LIFT‐AMFS system ensures high‐throughput sorting with minimal cell damage, yielding more stable RNA transcription profiles.

The cell‐friendly nature of this system can be attributed to several key factors: First, the innovative DSMF design facilitates rapid CTC enrichment under low shear stress, preserving cellular integrity while maintaining cells in a moist environment; Second, the design ensures that cells are positioned away from the metal layer surface during chip inversion, eliminating the risk of laser‐induced damage; Third, the noncontact retrieval method allows for high‐throughput sorting without direct physical interaction, minimizing the potential risk of cell damage. These advantages make LIFT‐AMFS a powerful tool for single‐cell analysis, particularly in the context of tumor cell heterogeneity\ studies.

## 3 Conclusion

In this study, a LIFT‐AMFS was successfully developed and validated for label‐free enrichment and retrieval of rare cells, such as CTCs, at the single‐cell level from undiluted human blood. The system, which integrates size‐based microfiltration with a nanosecond pulsed laser for precise single‐cell sorting, represents a significant advancement in the preparation of single cells. This system has shown promising results, with a capture efficiency of 88% and a high processing speed of up to 15.0 mL min^−1^ during the CTC enrichment stage, along with a single‐cell yield exceeding 95% in the retrieval stage.

Compared to existing platforms for single rare cell sorting (Table S5, Supporting Information), the LIFT‐AMFS presents several distinct advantages, such as label‐free enrichment, high cell viability, and excellent single‐cell yield, all without the need for sample preprocessing. These features render the LIFT‐AMFS particularly advantageous for single CTC sorting. Additionally, all these features are critical for connecting the system to downstream analyses at the single CTC level. LIFT‐AMFS effectively retrieves single cells with high viability, facilitating direct downstream analysis, including single‐cell ex vivo culture and RNA‐Seq. Furthermore, the system facilitates reliable correlation analysis between samples and differential gene expression analysis, highlighting its potential as a versatile tool for advancing single‐CTC research and applications.

In forthcoming studies, the duration of the moist environment will be extended through microfluidic technology. This will be achieved, for instance, by packaging the microfilters within carefully designed closed microcavities, to better preserve cell viability during LIFT sorting. Moreover, this platform could be used in clinical research to enhance the analysis of genetic material and the interpretation of rare cell heterogeneity, thereby broadening its application in key scientific areas.

## 4 Experimental Section

### DSMF Operation

For system assembly, the DSMF was mounted onto custom‐designed polycarbonate holders, which were fabricated via injection molding (Figure S10, Supporting Information). The employed assembly process, utilizing transparent materials, is straightforward and allows for real‐time observation of the processing progress and sample status. Unlike previous systems that used polymethyl methacrylate gadgets^[^ ^43^ ^]^ and ring magnet‐assisted sealing methods,^[^ ^44^ ^]^ the proposed assembly offers enhanced operational convenience and a more aesthetically pleasing design. The ease of assembly and real‐time visibility provided by the transparent materials constitute significant improvements over earlier designs, facilitating more efficient and effective CTC enrichment and retrieval.

### Cell Culture and Sample Preparation

Two human lung cancer cell lines (A549‐mCherry and PC‐9) were used as models to mimic actual CTCs in the following validation and optimization studies. The A549‐mCherry cell line was purchased from the Cell Resource Center, Peking Union Medical College. The PC‐9 cell line, obtained from The Second Hospital of Dalian Medical University, was stably transfected with a green fluorescent protein (GFP). The A549‐mCherry cells were cultured with McCoy's 5A modified Medium (Gibco, ThermoFisher, USA) with 10% FBS (Gibco, ThermoFisher, Australia) and 1% penicillin‐streptomycin (Gibco, ThermoFisher, USA) at 37 °C and 5% CO_2_. The PC‐9 cells were cultured in RPMI 1640 Medium (Gibco, ThermoFisher, USA) with 10% FBS and 1% penicillin/streptomycin at 37 °C and 5% CO_2._

Whole blood for cell line spiking studies was obtained from healthy volunteer donors. Blood samples from lung cancer patients were collected at the Second Affiliated Hospital of Dalian Medical University (Dalian, China), with clinical information summarized in Table S2 (Supporting Information). Informed consent was obtained from all the healthy donors and lung cancer patients before the collection of samples. This study was approved by the Ethics Committee of the Second Affiliated Hospital of Dalian Medical University (2023‐128). Blood samples were stored in ethylene diamine tetraacetic acid coated vacutainer tubes (BD Vacutainer) and processed within 24 h. Simulated blood samples for the optimization and validation studies were prepared by spiking 500–100000 tumor cells into the healthy donor blood.

### CTC Identification

CTCs were identified by modified immunostaining protocols. Cell fixation and permeabilization were removed to preserve cell viability. Briefly, after capturing the CTCs onto the DSMF, the cells were blocked in situ with 5% FBS in PBS for 30 min at room temperature. Subsequently, cells were simultaneously incubated with Alexa Fluor 555 conjugated cytokeratin 7 antibody (Abcam, UK) and Alexa Fluor 488 conjugated CD45 antibody (Abcam, UK) for 20 min. After immunofluorescence staining, the DSMF was washed with PBS (Gibco, Thermo Fisher, USA) and the nuclei were stained with Hoechst 33342 for 10 min. The CK7+ / CD45‐ / Hoechst+ expression was the CTCs, while the CK7‐ / CD45+ / Hoechst+ phenotype was confirmed to be the WBCs.

### DSMF Enrichment Performance Toward Rare Tumor Cells

The capture efficiency of the DSMF toward tumor cells was evaluated by spiking tumor cells into PBS and whole blood samples of healthy donors. More specifically, 10^4^ PC9‐GFP cells were spiked into 10 mL PBS to validate the processing capabilities for large‐volume samples. Before enrichment, the microfilter holders were assembled and supported by a 50 mL falcon tube. Then, 1 mL of 75% ethanol was loaded to wet the devices, followed by washing with 3 mL of PBS to remove residual ethanol. Subsequently, the sample was loaded and passed through the microfilter driven by gravity, which was followed by washing with 3 mL of PBS to elute unbound blood cells. Next, the capture efficiency of rare tumor cells from whole blood was characterized using a simulated blood sample (containing 500 PC9‐GFP cells in 1 mL undiluted whole blood) as a model. After enrichment, the cells captured across the filtration area of the microfilter were scanned under fluorescence microscopy (DFC9000 GT, Leica, Germany) and counted using the Image J software (National Institutes of Health, USA). To further assess the capture performance of DSMF with lower tumor cell concentrations, small numbers of PC9‐GFP cells (≈10, 50, 100 cells per mL) were spiked into PBS and whole blood under optimal capture conditions (design 2). The addition of 100 cells was performed via serial dilution, while ≈10 and 50 cells were introduced using the microdroplet‐based spiking method combined with a hydrophobic substrate. For precise cell addition, a glass slide coated with a 10× diluted Teflon solution (FC40), air‐dried to render it hydrophobic, and a small volume of microdroplet containing cells was carefully pipetted onto the slide. The number of cells in each droplet was confirmed under fluorescence microscopy (CKX53, Olympus, Japan) before adding them to the sample. After filtration, captured tumor cells on the microfilter were counted on the fluorescence microscopy. All experiments were repeated three times.

The capture efficiency was determined using the waste statistics method. The waste microfilter, characterized by a single layer with smaller micropore sizes (4.73 ± 0.05 µm), was used to collect the uncaptured nucleated cells. Following the enumeration of stained cells captured by the DSMF and the waste single‐layer microfilter, the capture efficiency was calculated as follows:

(1) $$\mathrm{Capture}\;\mathrm{efficiency}\;\left(\%\right)=\frac{N_{c}}{N_{c}+N_{cw}}\;\times 100\%$$

where *N_c_* represents the number of cells captured on the DSMF and *N_cw_* denotes the number of captured cells on the waste microfilter. The sample processing speed was investigated using six different microfilter designs (Table S1, Supporting Information). More than three experiments were conducted for each design and the average value was taken. The processing speed was calculated as the ratio of the whole blood volume (6 mL) to the total duration of the enrichment process.

The viability of the cells captured on the DSMF was categorized into short‐term and long‐term viability. The microfilters were first subjected to decontamination procedures involving immersion in 75% alcohol for 30 min followed by exposure to UV light for an additional 30 min. Short‐term viability analysis was conducted utilizing the staining of Calcein‐AM (for labeling live cells) and ethidium homodimer‐1 (EthD‐1, for labeling dead cells). First, 10^5^ PC‐9 cells suspended in 10 mL PBS were enriched using the DSMF. Afterward, the microfilter was transferred to a 35 mm cell culture plate and soaked in a solution containing Calcein‐AM and EthD‐1 for 30 min at room temperature. Subsequently, the live and dead cells on the microfilter were observed under a spinning disk confocal microscope (S3000, Hooke Instruments Ltd., China) and quantified using the Image J software. Concurrently, cells subjected to the same conditions without filtration were also stained with the same staining solution and imaged under the microscope as the control group. Cell viability was determined as follows:

(2) $$\mathrm{Cell}\;\mathrm{viability}\;\left(\%\right)=\frac{N_{l}}{N_{l}+N_{d}}\times 100\%$$

where *N_l_* and *N_d_* are the numbers of live and dead cells, respectively. Each experiment was performed at least 3 times. To further evaluate the post‐enrichment long‐term viability of cells, the cells captured on the microfilter (design 5 in Table S1) were cultured in a 35 mm cell culture plate with 3 mL RPMI 1640 culture medium in situ. Images of the cells were captured on days 1, 3, and 5 using confocal fluorescence microscopy. In this step, the cells were illuminated by Calcein‐AM staining (1 µg mL^−1^ in culture medium) for 15 min.

### Single Cell Retrieval Performance using LIFT‐AMFS

A single target cell was retrieved from the DSMFs bonding with the 50‐nm‐thick TiN glass substrate. A 5 ns pulsed laser vaporized the TiN layer to release the target cell with minimal damage. Different laser energy levels ranging from 100 to 500 nJ were tested on the different microfilters by spiking A549‐mCherry cells into PBS and whole blood. The experiments for each design were repeated over 20 times at each laser energy level. The retrieval efficiency and single‐cell yield were calculated as follows:

(3) $$\mathrm{Retrieval}\;\mathrm{efficiency}\;\left(\%\right)=\frac{N_{s}}{N_{t}}\;\times 100\%$$

(4) $$\mathrm{Single}-\mathrm{cell}\;\mathrm{yield}\;\left(\%\right)=\frac{N_{ssc}}{N_{s}}\;\times 100\%$$

where *N_t_* represents the total number of experiments conducted under each laser energy level, *N_s_* indicates the number of successful cell retrievals, and *N_ssc_* denotes the number of retrieved single cells.

The single‐cell retrieval process was simulated utilizing the heat transfer module of COMSOL Multiphysics. A double‐layer micropore structure was constructed to represent the physical model, wherein the thermal energy generated by the interaction of the laser with TiN was transferred to the liquid domain within the micropore structure, leading to temperature distribution via diffusion. More specifically, the physical model was configured with the following parameters: laser energy of 300 nJ, micropore size of 10 µm for the lower layer 25 µm for the upper layer, and TiN thickness of 50 nm.

The viability of the retrieved cells was evaluated through long‐term culturing. Prior to each experimental session, the microfilters were subjected to a rigorous decontamination protocol to ensure the absence of any potential contaminants. This involved soaking the filters in a 75% ethanol solution for 30 min, followed by a 30‐min exposure to ultraviolet (UV) radiation. Next, 10^5^ PC‐9 cells suspended in 10 mL PBS were enriched using the microfilter (design 6 in Table S1, Supporting Information). Subsequently, the target cells were picked up using the single‐cell sorter based on LIFT (PRECI SCSF, Hooke Instruments Ltd., China) and seeded into a 96‐cell culture plate. The retrieved cells were then cultured using 350 uL RPMI 1640 culture medium supplemented with 20% FBS. To monitor the progression of cell proliferation, images of the cells were captured on days 0 (immediately after microfiltration), 3, and 5 via a spinning disk confocal microscope.

### Single‐Cell RNA‐Seq of Retrieved Cells

After capturing and identifying single target cells from whole blood, ten single PC‐9 cells were sorted using both the LIFT‐AMFS and the micromanipulation system. The sorted cells were immediately transferred into PCR tubes containing a reaction buffer, including lysis buffer and RNase inhibitor. The lysates containing single‐cell RNA were processed using the SMART‐Seq v4 Ultra Low Input RNA Kit (Clontech, USA) for reverse transcription and cDNA amplification. The quantity and quality of the amplified cDNA products were assessed using Qubit 4.0 (Thermo Fisher, USA) for total amount measurement and Agilent 5300 (Agilent, USA) for fragment distribution analysis. Subsequently, the cDNA library for sequencing was prepared according to the Nextera XT DNA Library Prep Kit (FC‐131‐1096) protocol. Lastly, the resulting single‐cell cDNA libraries were sequenced using the NovaSeq Xplus platform (Illumina, USA) to generate high‐quality sequencing data.

### RNA Sequencing Data Analysis

The quality of the raw single‐cell RNA‐Seq data was first assessed using Fastp (v.0.23.4), which removed adapter sequences and low‐quality reads. Then, the cleaned reads were aligned to the human genome reference (hg38) using Hisat2 (v.2.2.1) to ensure accurate mapping. After alignment, the expression levels of genes were quantified using RSEM (v.1.3.3), allowing for the detailed analysis of the gene expression profiles at the single‐cell level.

### Statistical Analysis

Experimental data was presented as mean ± SD unless otherwise specified. Sample size (n) was included in the figure legends. Statistical analyses were performed using OriginPro 2018 software (OriginLab, Northampton, USA).

## Conflict of Interest

The authors declare no conflict of interest.

## Author Contributions

Q.X. and Y.W. contributed equally to this work. Q.X., Y.W., Q.W., B.L., and W.W. supervised the project and wrote the manuscript, with contributions from the other authors. Q.X. and Y.W. conceived the project and designed the experiments. Q.X., S.D., H.X., and F.M. fabricated the devices. Y.W. and H.W. conducted numerical simulations. Y.X., Z.X., and Y.X. helped with the library preparation and RNA sequencing. K.Z. and H.L. provided the necessary help in the experiments on the viability of the captured cells. Y.X., Y.Z., and X.M. participated in the design of the illustrations. All authors have given approval to the final version of the manuscript.

## Supporting information



Supporting Information

Supplemental Movie 1

Supplemental Movie 2

## Data Availability

The data that support the findings of this study are available from the corresponding author upon reasonable request.
