# Supplementary material for: High‐Viability Circulating Tumor Cells Sorting From Whole Blood at Single Cell Level Using Laser‐Induced Forward Transfer‐Assisted Microfiltration
Source: Adv Sci (Weinh). 2025 Jan 27;12(18):2414195. doi: 10.1002/advs.202414195 (PMC12079465; doi:10.1002/advs.202414195)
Supplement: Supplementary file 1 — Supporting Information [file ADVS-12-2414195-s002.docx]

Supporting Information

High-Viability Circulating Tumor Cells Sorting from Whole Blood at Single Cell Level Using Laser-Induced Forward Transfer-Assisted Microfiltration

Qingmei Xu^1,2^†, Yuntong Wang^3,4^†, Songtao Dou^1^, Yang Xu^5,6^, Zhenhe Xu^7^, Han Xu^1^, Yi Zhang^1^, Yanming Xia^8^, Ying Xue^9^, Hang Li^9^, Xiao Ma^10^, Kunlong Zhang^9^, Huan Wang^3,4^, Fengzhou Ma^1,10^, Qi Wang^5,6^*****, Bei Li^3,4^*****, Wei Wang^1,11,12^*****

^1^School of Integrated Circuits, Peking University, Beijing, 100871, China

^2^Department of Electrical Engineering, Taiyuan Institute of Technology, Taiyuan, 03008, China

^3^Changchun Institute of Optics, Fine Mechanics and Physics, Chinese Academy of Sciences, Changchun, 130033, China

^4^University of Chinese Academy of Sciences, Beijing, 100049, China

^5^Cancer Translational Medicine Research Center, The Second Affiliated Hospital of Dalian Medical University, Dalian, 116027, China

^6^Department of Respiratory Medicine, The Second Affiliated Hospital of Dalian Medical University, Dalian, 116027, China

^7^State Key Laboratory of Bioreactor Engineering, Shanghai Frontiers Science Center of Optogenetic Techniques for Cell Metabolism, East China University of Science and Technology, Shanghai, 200237, China

^8^Guangzhou National Laboratory, Guangzhou, 510320, China

^9^Hooke Laboratory, Changchun, 130033, China

^10^Hangzhou Branemagic Medical Technology Co. Ltd., Hangzhou, 310021, China

^11^National Key Laboratory of Advanced Micro and Nano Manufacture Technology, 100871, Beijing, China

^12^Beijing Advanced Innovation Center for Integrated Circuits, Beijing, 100871, China

†The two authors contributed equally to this work.

* **Corresponding Author ：**Wei Wang (Email: [w.wang@pku.edu.cn](mailto:w.wang@pku.edu.cn))

* **Corresponding Author ：**Bei Li (Email: [beili@ciomp.ac.cn](mailto:beili@ciomp.ac.cn) )

* **Corresponding Author ：**Qi Wang (Email: wqdlmu@dmu.edu.cn )


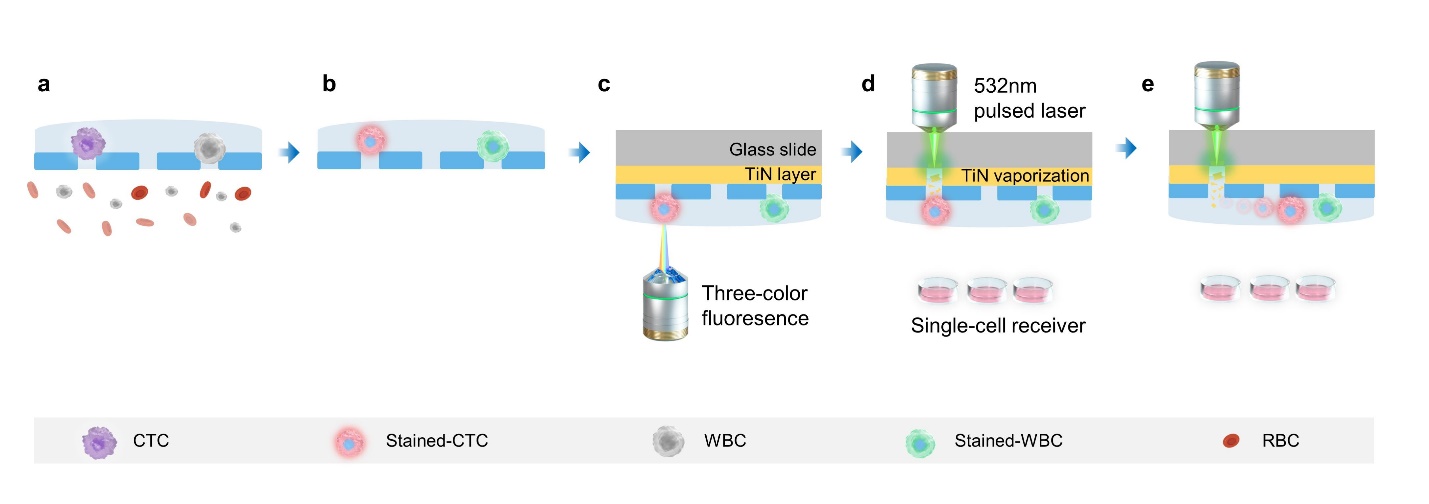


Figure S1. Single-CTC enrichment and retrieval using Parylene C-based single-layer microfilter. a) CTC enrichment from whole blood via single-layer microfilter. b) CTC identification using modified immunostaining protocols. c) CTC location by three-color fluorescence. d) CTC retrieval under pulsed laser. e) The retrieved cells could only move on the surface of the microfilter.


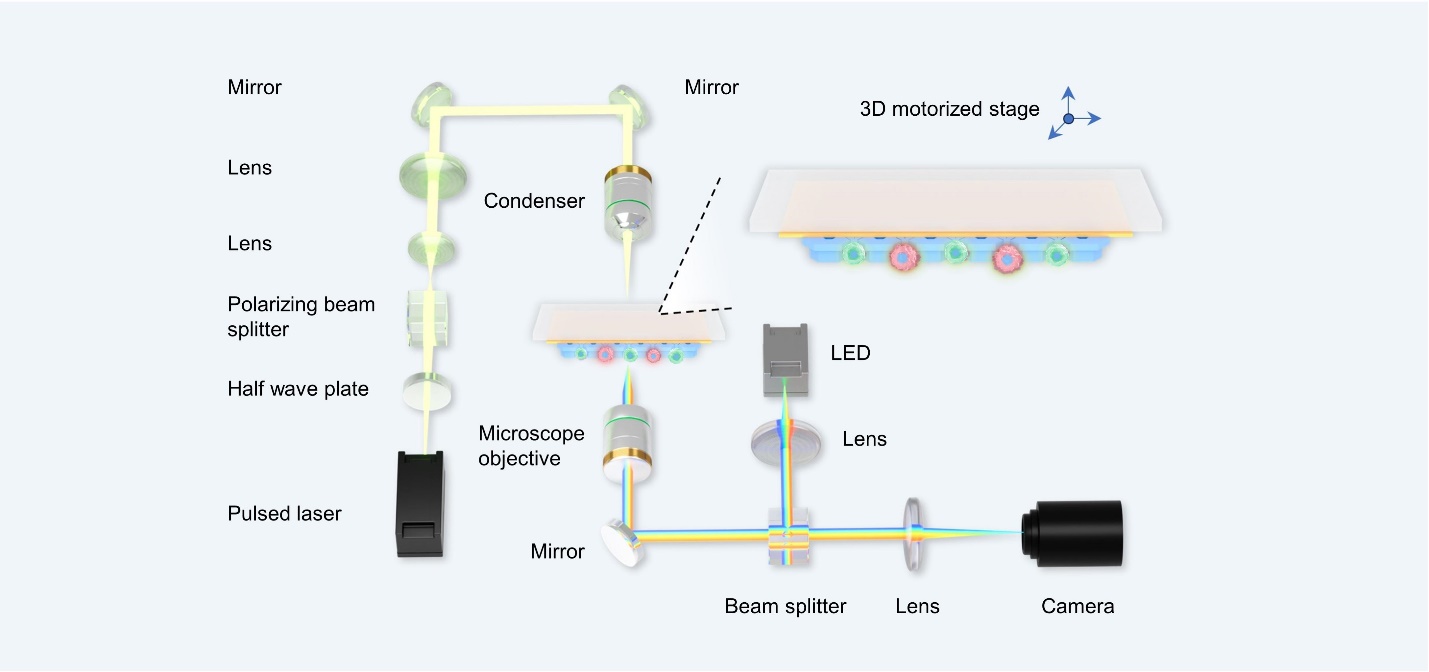


Figure S2. The schematic of the LIFT platform.


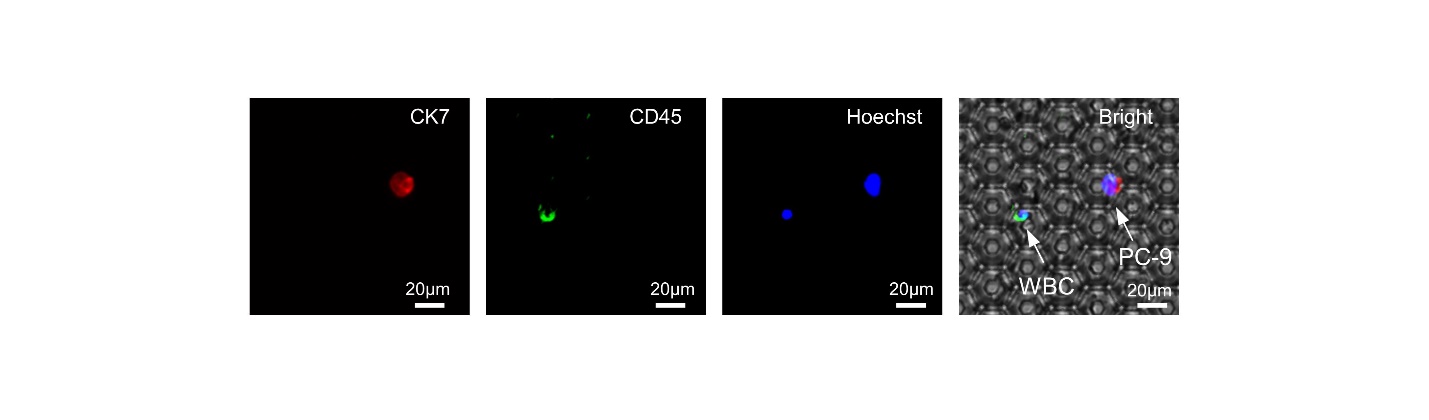


Figure S3. Identification of CTCs based on a live cell staining protocol using CK7 (a marker for CTC), CD45 (a marker for WBC) and DAPI (nuclear specific).


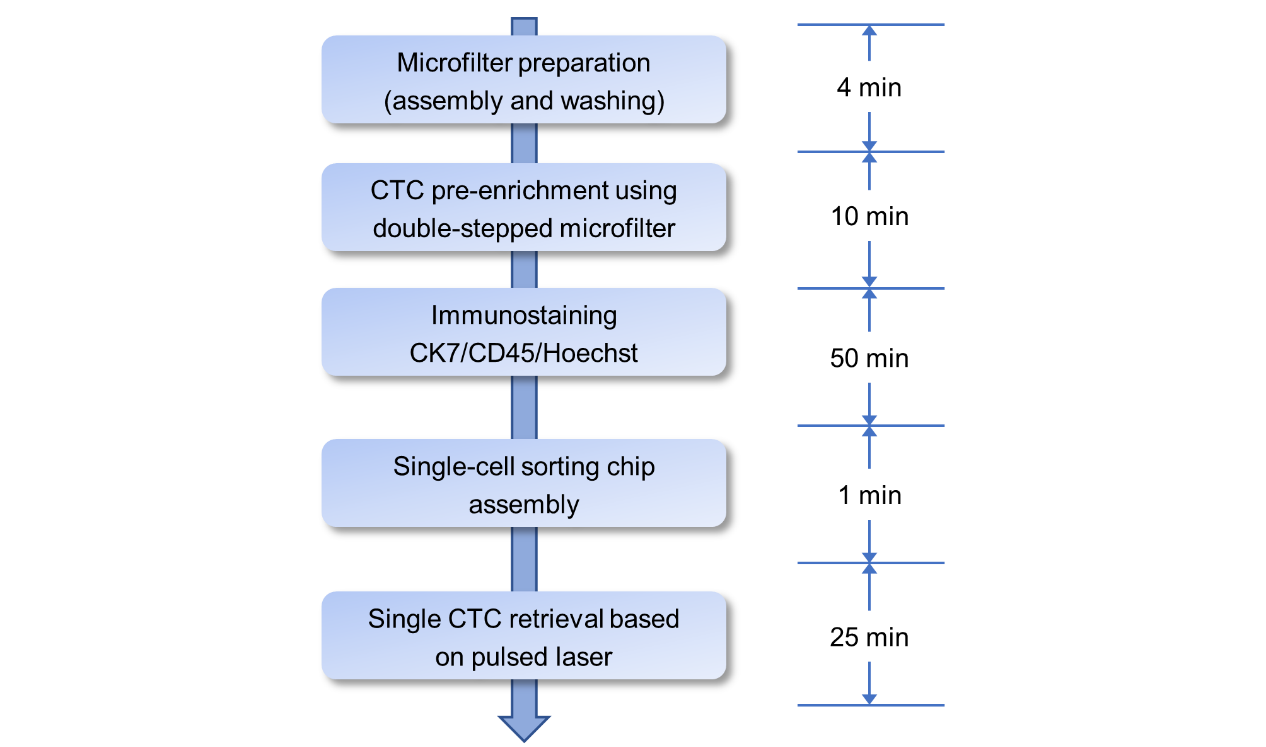


Figure S4. Complete workflow and operation time for each step in the LIFT-AMFS.


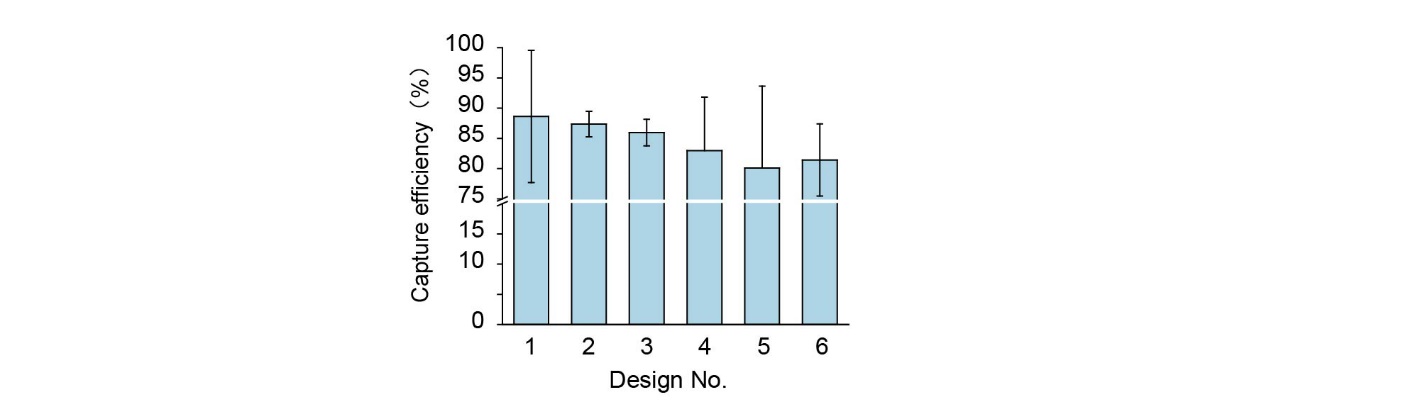


Figure S5. Capture efficiency of PC9-GFP cells in PBS with different microfilters. Data are presented as mean ± SD (n = 3).

***
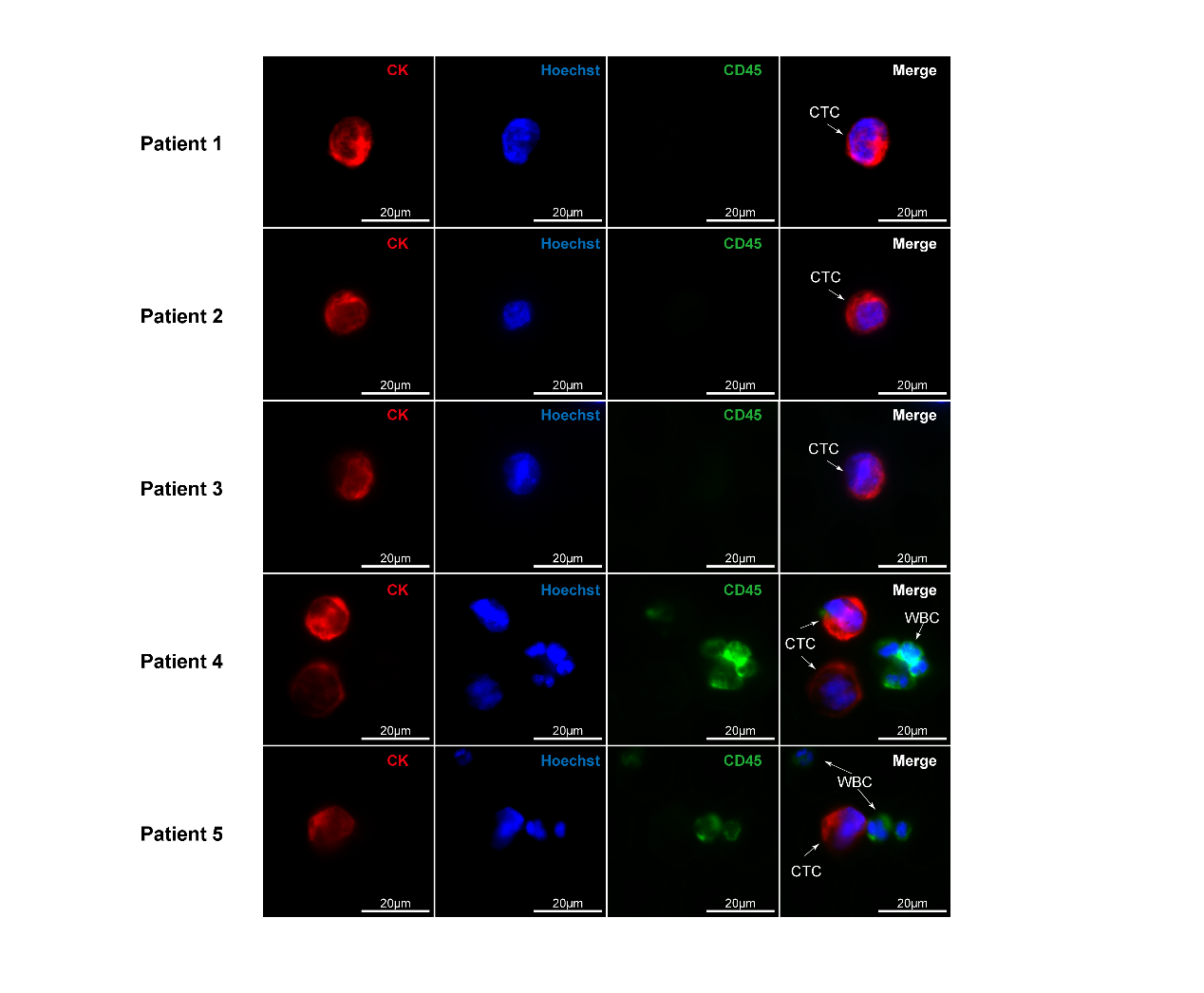
***

Figure S6. Typical images of the CTCs and WBCs on the DSMF in lung cancer patients.

**
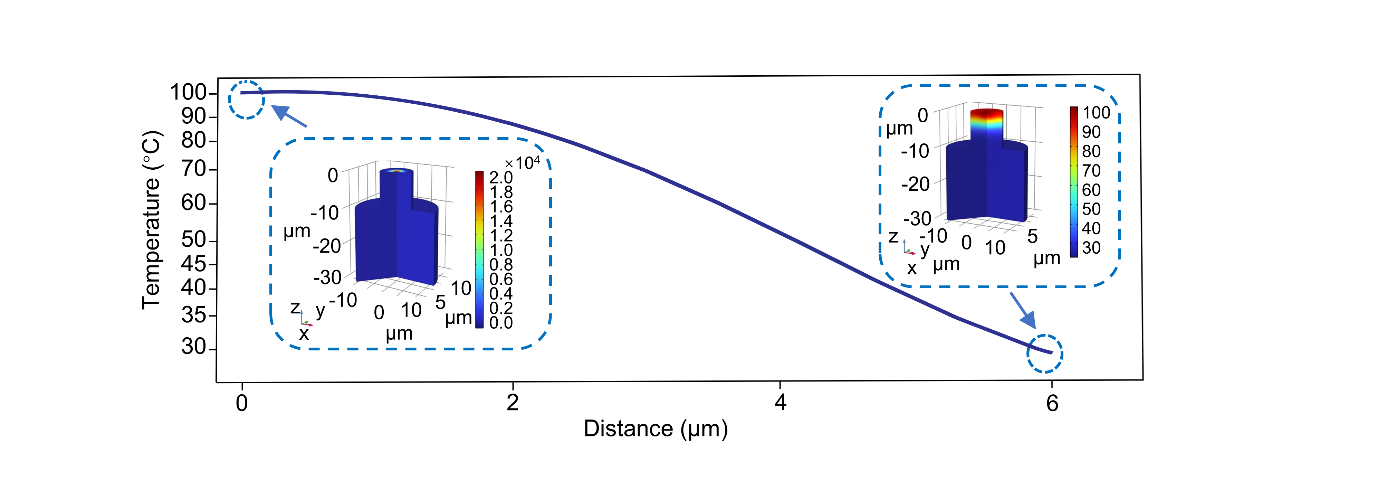
**

Figure S7. Spatial changes in temperature across the fluid domain at various distance intervals. The inset depicts the temperature distribution in the liquid induced by the laser energy during the CTC isolation using the DSMF.


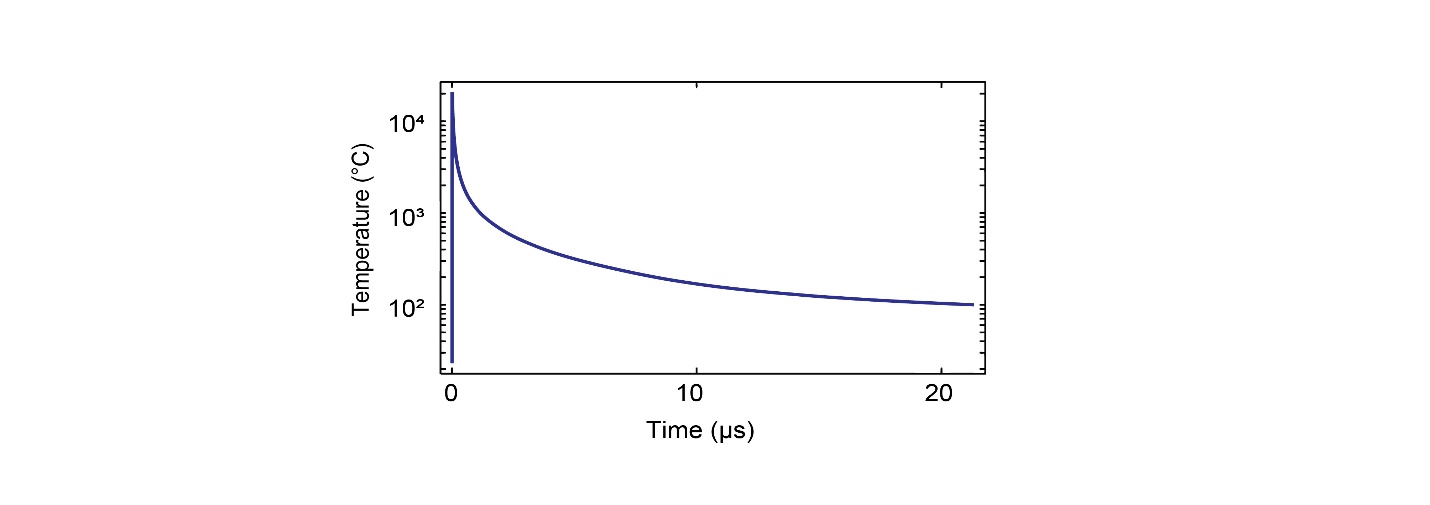


Figure S8. Temporal evolution of surface temperature fluctuations on the sacrificial layer subsequent to laser ejection.


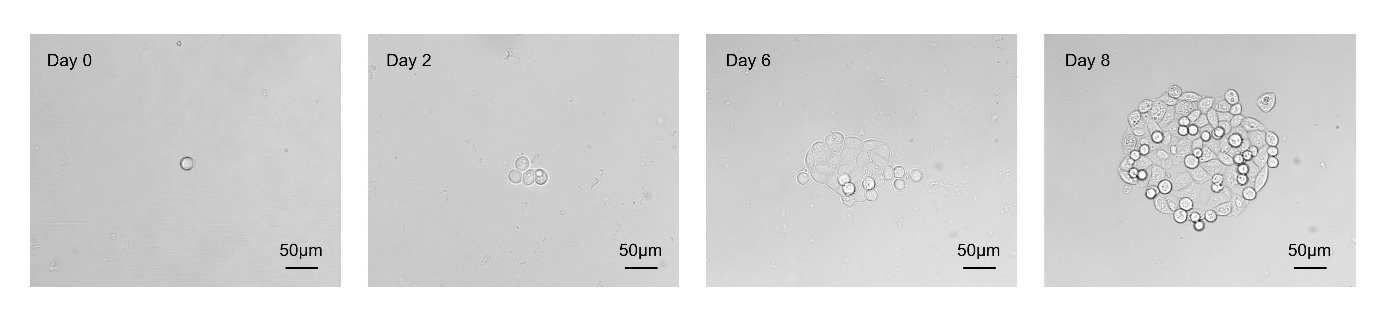


Figure S9. Optical microscopy images of retrieved tumor cells on days 0 (immediately after microfiltration), 2, 6, and 8.


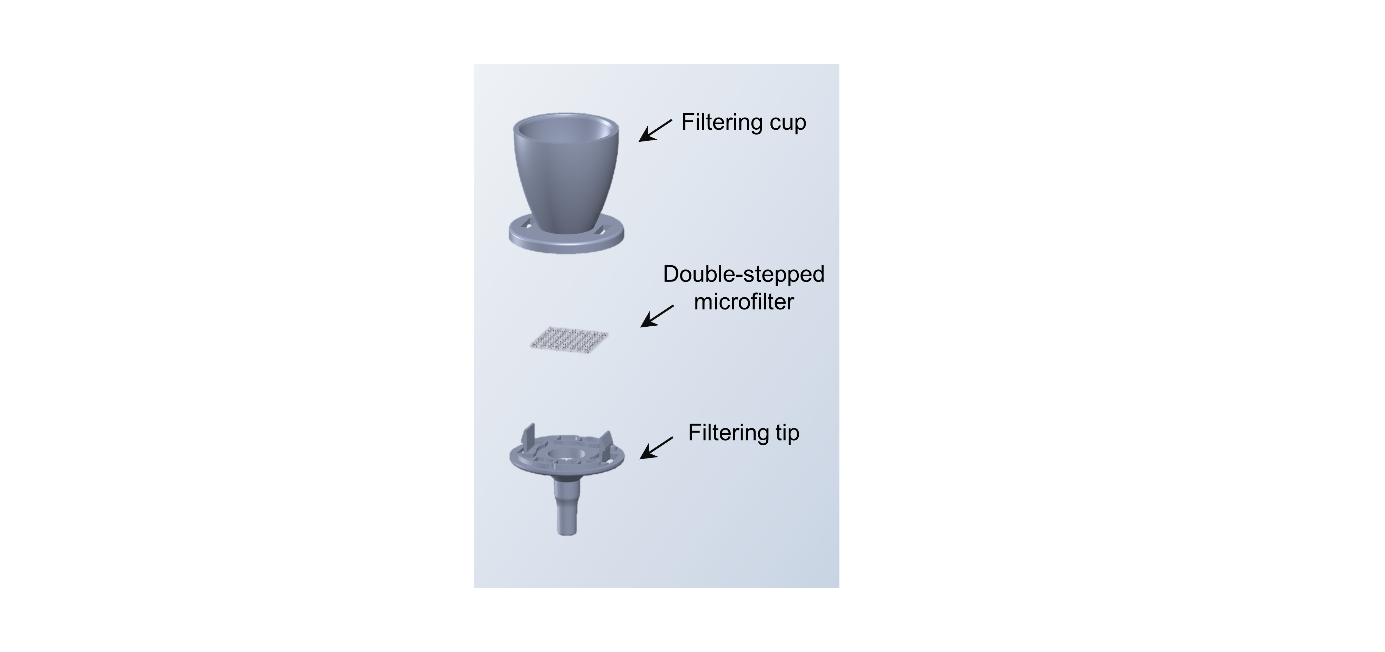


Figure S10. Assembly process of the DMSF via custom-designed polycarbonate holders.

Table S1. Parameters of double-stepped microfilter.

| **Design No.** | **Design value in mask (μm)** | | | | **Measured value after fabrication**  **(μm)** | | | | **Porosity (%)** | |
| --- | --- | --- | --- | --- | --- | --- | --- | --- | --- | --- |
|  | **D** | **d** | **S** | **s** | **D** | **d** | **S** | **s** | **Upper-layer** | **Lower-layer** |
| 1 | 15 | 8 | 4 | 10.06 | 14.15±0.12 | 6.64±0.08 | 3.79±0.16 | 10.38±0.20 | 58.46 | 16.64 |
| 2 | 20 | 8 | 4 | 14.39 | 19.07±0.11 | 7.06±0.09 | 3.97±0.16 | 14.30±0.26 | 66.00 | 10.56 |
| 3 | 25 | 8 | 4 | 18.72 | 23.80±0.13 | 6.79±0.11 | 3.50±0.22 | 18.44±0.34 | 71.24 | 7.30 |
| 4 | 15 | 10 | 4 | 8.33 | 14.42±0.08 | 9.36±0.08 | 3.65±0.20 | 8.20±0.16 | 58.46 | 25.98 |
| 5 | 20 | 10 | 4 | 12.66 | 19.17±0.09 | 9.32±0.09 | 3.89±0.17 | 12.50±0.34 | 66.00 | 16.50 |
| 6 | 25 | 10 | 4 | 16.99 | 23.72±0.16 | 9.16±0.09 | 4.35±0.29 | 17.08±0.42 | 71.24 | 11.40 |

Table S2. Information and the count of CTCs in lung cancer patients.

| **Patient No.** | **Age** | **Sex** | **Pathology type** | **CTC count** |
| --- | --- | --- | --- | --- |
| 1 | 54 | Female | Adenocarcinoma | 3 |
| 2 | 59 | Male | Adenocarcinoma | 2 |
| 3 | 48 | Male | Adenocarcinoma | 6 |
| 4 | 73 | Male | Squamous cell carcinoma | 4 |
| 5 | 70 | Female | Adenocarcinoma | 11 |

Table S3. Single-cell yield of retrieval target cells from whole blood using DSMF.

| **Design No.** | **Single-cell yield (%)** | | | | |
| --- | --- | --- | --- | --- | --- |
|  | **Laser energy (nJ)** | | | | |
|  | **100** | **200** | **300** | **400** | **500** |
| 1 | - | 100 | 100 | 100 | 100 |
| 2 | - | 100 | 100 | 100 | 100 |
| 3 | - | 100 | 100 | 100 | 100 |
| 4 | 100 | 100 | 95 | 95 | 100 |
| 5 | - | 100 | 100 | 100 | 95 |
| 6 | - | 100 | 95 | 100 | 100 |

Table S4. Single-cell RNA-Seq data quality using LIFT-AMFS.

| Sample No. | Raw reads | Clean reads | Error  rate (%) | Q20(%) | Q30(%) | GC  content (%) |
| --- | --- | --- | --- | --- | --- | --- |
| LAMF1 | 51,919,580 | 50,542,758 | 0.012 | 98.65 | 96.10 | 49.89 |
| LAMF2 | 54,141,932 | 52,738,314 | 0.012 | 98.67 | 96.14 | 49.26 |
| LAMF3 | 58,445,020 | 57,048,234 | 0.012 | 98.75 | 96.31 | 49.95 |
| LAMF4 | 52,867,750 | 51,534,300 | 0.012 | 98.70 | 96.22 | 49.81 |
| LAMF5 | 45,824,896 | 44,560,574 | 0.012 | 98.58 | 95.92 | 50.17 |

Table S5. Comparison of different microfluidic-based platforms for single rare cell sorting from whole blood.

| **Method** | | **Throughput** | | | **Recovery** | **Single-cell yield** | **Advantages** | **Disadvantages** | **Ref.** |
| --- | --- | --- | --- | --- | --- | --- | --- | --- | --- |
| **Rare cell enrichment** | **Rare cell retrieval** | **Rare cell enrichment** | **Rare cell retrieval** | **Rare cell identification (Immunofluorescence staining)** |  |  |  |  |  |
| Immune affinity-based nanopillars | Laser microdissection | 1 mL/h | 5-30 min per cell | 60 min | >70% | N.A. | High sensitivity and specificity | Nonspecifically contamination from background cells, low processing efficiency | (35) |
| Immune affinity-based micropillars | Pulse ablation | 1 mL/h | 1 second per cell | <3 h | 95.9-98.7% | 77.30% | High recovery rate, non-contact retrieval method, cell-viability friendly | Nonspecifically contamination from background cells | (38) |
| Density gradient Centrifugation | Micromanipulation | 2.5h | | N.A. | 58.3-81.9% | 40.30% | Simple operation, lable-free method | Potential cell damage, low processing efficiency | (32) |
| Immune affinity-based micropillars | Fluorescence-activated cell sorting | 90 μL/min | ~6 min per cell | 60 min | ~75-87% | N.A. | Minimum cell loss and and transfer | Low throughput | (21) |
| Size-based microfiltration | Laser-induced forward transfer | 2.3-15 mL/min | 1 second per cell | 50 min | 76.2-88.3% | 95.0-100% | Non-contact retrieval method, lable-free method, high single-cell yield, cell-viability friendly, no sample preparation | Duration of moist liquid environment during cell retrieval | This study |
